# An ambient temperature-stable antitoxin of nine co-formulated antibodies for botulism caused by serotypes A, B and E

**DOI:** 10.1371/journal.pone.0197011

**Published:** 2018-05-10

**Authors:** Mingxiang Li, Dennis Lee, Chidi R. Obi, Joel K. Freeberg, Shauna Farr-Jones, Milan T. Tomic

**Affiliations:** 1 XOMA Corp., Berkeley, CA, United States of America; 2 Department of Anesthesia and Perioperative Care, University of California, San Francisco, San Francisco, CA, United States of America; US Naval Research Laboratory, UNITED STATES

## Abstract

Safe and effective antitoxins to treat and prevent botulism are needed for biodefense. We have developed recombinant antibody-based therapeutics for botulinum neurotoxin (BoNT) serotypes A, B, and E. The mechanism of action of this antitoxin requires that three mAbs bind one toxin molecule to achieve clearance. Here we present a co-formulation of an antitoxin to the three most important serotypes. Combining these antibodies obviates the need to identify the serotype causing intoxication prior to drug administration, which would facilitate administration. The lyophilized powder formulation contains nine mAbs, three mAbs for each of the three serotypes (A, B, E). The formulation was stored as a liquid and lyophilized powder for up to one year, and characterized by binding affinity and multiple physicochemical methods. No significant increase in soluble higher order aggregates, cleavage products, or change in charge isoforms was measured after storage as a lyophilized powder at 50°C for one year. Furthermore, toxin-domain binding ELISA data indicated that each of the individual antibodies in the lyophilized drug product showed essentially full binding capability to their respective toxin domains after being stored at 50°C for one year. Physicochemical characterization of the formulation demonstrated the nine individual mAbs were remarkably stable. This work demonstrates feasibility of lyophilized, oligoclonal antibody therapies for biodefense with ambient temperature stability, that would facilitate stockpiling, distribution, and administration.

## Introduction

Botulinum neurotoxin (BoNT) is one of the six category A biothreat agents and therefore development of safe and effective antitoxins is a priority to protect from potential bioterror use [1,2]. The sequence diversity leading to multiple serotypes and subserotypes presents technical challenges for a pan-botulism antibody-based drug. Each of the eight known serotypes of BoNT requires a serotype-specific antitoxin due to the sequence diversity of the toxins. Eight immunologically-distinct groups of neurotoxins (BoNT/A-G and BoNT/HA) [3,4] differ by 35–68% at the amino acid level. Adding further diversity, multiple subserotypes exist for at least six of the eight known serotypes [5,6]. The subserotypes differ by 2.6 to 36% at the amino acid level and these differences can have dramatic impact on the binding and neutralization of toxin by monoclonal and polyclonal antibodies [6–9]. Three of the BoNT serotypes (A, B, E) cause approximately 97% of the cases of human botulism [10–12] and therefore present the most urgent need for drug development.

The numerous limitations of current equine-derived botulinum antitoxin, BAT, [13] has provided impetus for development of a recombinant antitoxin that is consistent, safer, more effective, and is renewable. Therefore, the National Institute of Allergy and Infectious Disease (NIAID) has funded discovery and development of monovalent human monoclonal antibody (mAb)-based antitoxins that neutralize BoNT serotypes A, B, and E (BoNT/A, BoNT/B, and BoNT/E). Each antitoxin consists of a combination of three recombinant mAbs binding non-overlapping BoNT epitopes with very high affinity. The mAbs in each anti-serotype combination have been selected to be cross reactive toward all known subserotypes for that serotype [14,15] and unpublished. Using these serotype-specific antitoxins, a lyophilized nine-mAb product to prevent and treat botulism from exposure to BoNT/A, /B, and /E has been developed.

Macaque [16], and humanized macaque [16,17] mAbs neutralizing BoNT/ ABE have been reported by others. A discussion of the relative properties of the European AntibotABE Framework Program [16] and those generated by Marks et al., has been published [15].

The oligoclonal strategy of combining three mAbs that bind non-overlapping epitopes leads to highly potent BoNT neutralization [18] due to multiple mechanisms including first pass hepatic clearance of the immune complexes. Three mAbs are needed as no single mAb neutralizes BoNT/A with a potency greater than 1000 mouse LD_50_/mg of antibody [18,19].

Here we report development of a nine mAb botulinum antitoxin prototype drug product consisting of nine mAbs to be administered by injection for the treatment and prevention of botulism resulting from exposure to BoNT serotypes A, B, or E [20]. The lyophilized BoNT 9ABE antibody product (anti-BoNT 9ABE) was designed to have long-term ambient stability suitable for stockpiling, does not require a cold chain, and that will address the three BoNT serotypes that cause approximately 97% of human botulism. Each of the nine mAbs in the mixture was engineered to have properties of high affinity, broad subserotype specificity, and inherent stability. Sequence elements known to be prone to chemical degradation by oxidation, deamidation, hydrolytic cleavage and fragmentation were substituted to eliminate reactivity that could lead to instability. An identical antibody constant (Fc) region was used for all mAbs to impart common biochemical properties critical for formulation optima of pH and ionic strength. Multiple antibody variants of each antitoxin serotype domain specificity were screened to minimize propensity for aggregation while retaining unique attributes of charge and hydrophobicity to facilitate analytical separation.

While a stable liquid formulation is an important first step in development of an oligoclonal recombinant antitoxin, a room-temperature-stable product is highly desirable to eliminate the cold chain requirement, simplifying storage and distribution. The anti-BoNT 9ABE mixture was subjected to a conservative lyophilization cycle followed by storage at refrigerated, stress and accelerated temperatures. Lyophilized anti-BoNT 9ABE formulations showed minimal changes with respect to aggregation and charge isoform distribution as well full retention of toxin binding activity after twelve months at 50°C, a significant improvement compared to liquid formulations. Stability testing suggests a room-temperature-stable trivalent drug product is feasible.

While others have studied co-formulation of antibodies [21],[22], to our knowledge, co-formulation of nine antibodies with long-term ambient-temperature stability is unprecedented. Oligoclonal antibodies for cancer treatment are being developed ([23] and reviewed in [24]), however, the unique requirements for biodefense have stimulated our efforts to produce antibody cocktails that have high levels of stability.

## Materials and methods

### Monoclonal anti-BoNT antibodies

We previously developed nine recombinant IgG1 monoclonal antibodies to neutralize BoNT serotypes A, B and E. MAbs were generated from humans immunized with pentavalent toxoid via phage or yeast antibody libraries [15,18,20,25]. Lead antibodies were characterized with respect to affinity for the different BoNT/A, B, or E subtypes and then optimized for binding using in vitro affinity maturation and yeast display [7,20,25,26]. The mAbs comprising anti-BoNT 9ABE have a common human light and heavy chain constant region to facilitate co-formulation. Each mAb was expressed in stably transfected Chinese hamster ovary (CHO) cell lines, purified, and then combined to produce the equimolar nine antibody mixture. The mAbs to serotype A have been previously described [27].

### Sample preparation

Each of the nine mAbs comprising anti-BoNT 9ABE (individually formulated in 10mM succinate buffer pH 6.0 containing 142 mM arginine, and 0.005% Tween 90) were concentrated with VivaSpin centrifugation device, molecular weight cut off 10 kDa (Vivaproducts Inc., Littleton, MA, USA) to approximately 40–100 mg/mL. The concentration of each antibody was adjusted to 30 mg/mL with the formulation buffer (10mM sodium succinate, 142 mM L-arginine, pH 6.0) [28] to obtain the drug substance. The solution was then filtered with a 0.45 um filter. This formulation was the same as that developed for the three-antibody mixture anti-BoNT A [27]. One percent (w/v) Polysorbate 20 (PS20) aqueous solution was added to the anti-BoNT 9ABE drug substance to obtain a final PS20 concentration of 0.01% as the formulated solution for lyophilization.

The formulated anti-BoNT 9ABE was aliquoted into 3 ml glass vials (1 ml per vial), and two-leg rubber lyo stopper was partially inserted, and vials were lyophilized for 44.5 hours with shelf loading on a Genesis 25XL, Virtis Lyophilizer controlled by Encore Software (SP Scientific, Warminster, PA, USA). Temperature started at 5°C, freezing to -50°C over 30 minutes, held at -50°C for 3 hours, then vacuum was applied to 200 mTorr. Temperature was raised to -30°C over 40 minutes. Samples were held for 15 hours each at -30°C for 17 hours and -20°C for 13.6 hours and then at 25°C for 8.3 hours at 200 mTorr. Vials were stoppered just before the chamber pressure reached atmospheric pressure and capped with aluminum overseals. Liquid anti-BoNT 9ABE drug substance stored at 5°C was used as a control. Five to six vials of each formulation were placed at each of the three temperature stability chambers (5°C, 30°C, and 50°C) to be tested at various times for stability assays.

### Analytical methods

Antibody concentrations were determined with UV absorption at 280 nm (A_280_) using theoretical extinction coefficients calculated using proprietary software SeqAgent^TM^ [29]. All HPLC analyses were conducted with an Agilent 1100 or 1200 system (Agilent Technologies, Inc., Santa Clara, CA, USA). The data acquisition and peak integration analysis were conducted with the ChemStation software (Rev. B.03.02). The injection amount was 60–300 μg. The detection was by UV at 280 nm and 214 nm.

Size exclusion- high-pressure liquid chromatography (SE-HPLC) was performed with a Zenix SEC-300 (7.8 x 300 mm) column (Sepax Technologies, Inc., Newark, DE, USA). The elution buffer was 20 mM sodium phosphate, 35 mM ammonium sulfate, 130 mM sodium chloride, pH 6.8. The flow rate was 0.7 mL/min and run time was 22 minutes. Peak integration was performed using ChemStation software (Rev. B.03.02).

Reverse phase-HPLC (RP-HPLC) was conducted with a Source 5RPC (4.6 x 150 mm) column (GE Healthcare, Little Chalfont, UK). The column temperature was 68°C during analysis. Elution buffer A was 0.075% trifluoroacetic acid (TFA) in water and elution buffer B 0.075% TFA in acetonitrile. The antibodies were eluted with gradients listed in in Table 1.

**Table 1:** Gradient profile for RP-HPLC elution profile.

Time (Minutes)	0	3	45	45.1	50	50.1	58	58.1
**% B**	36	36	40.2	70	70	36	36	36
**Flow rate (mL/min)**	0.4			0.8				0.4

Weak cation exchange-HPLC (WCX-HPLC) was conducted with the Dionex (Thermofisher Scientific Inc., Waltham, MA, USA) ProPac WCX-10 (4 x 250 mm) column at a flow rate of 1 mL/min. The elution buffer A was 10 mM sodium phosphate, pH 6.0 and buffer B 0.3 M sodium phosphate, pH 7.3. The antibodies were eluted with gradients listed in Table 2.

**Table 2:** Weak cation exchange-HPLC gradient profile.

Time (minutes)	0	30	40	50	60
**% B**	0	12	20	40	40

Differential scanning calorimetry (DSC) was conducted with NanoDSC (TA Instruments, New Castle, DE, USA) and analyzed with NanoAnalyze software. Sample concentrations were approximately 2 mg/mL of antibody.

Dynamic light scattering (DLS) analysis was conducted with a DynaPro Plate Reader (Wyatt Technologies Inc., Goleta, CA, USA). The diluted samples (~80 μL of 50-fold diluted in purified water) were loaded into Corning polystyrene 96-well half area plates. The auto-correlation function was collected for 5 seconds and 10 acquisitions were obtained for each sample at 25°C. Hydrodynamic radius, percentage polydispersity, and relative percent mass were determined using Dynamics 7.1.6.5 software by choosing the regularization mathematical simulation approach to fit the autocorrelation function.

ELISA was used to measure specific binding of antibodies to their respective BoNT A, B or E domains as previously reported [30,31]. The toxin domains, engineered to contain only single mAb epitopes, were expressed in *E*. *coli* and purified by affinity column chromatography. Domain proteins were coated onto 96-well microtiter plates. anti-BoNT 9ABE solution was added to the plates after serial dilution. A secondary anti-human Fc antibody conjugate with horseradish peroxidase was used for detection after which, the substrate 2,2'-azino-bis (3-ethylbenzothiazoline-6-sulphonic acid, ABTS) was added. Absorbance at 405 nm (A_405_) for various mAb concentrations was recorded and fitted to a four-parameter equation to give a sigmoidal curve using SoftMax Pro software v5.2 (Molecular Devices, LLC, San Jose, CA).

Capillary electrophoresis (CE-SDS) was performed on a ProleomeLab PA800 (Beckman Coulter, Inc., Brea, CA) fitted with a photo diode array detector. The capillary was the eCAP^TM^ bare fused silica, 50 μm x 30.2 cm. The samples included a 10 kDa internal standard and were heated at 70°C for 10 minutes in the presence of either 12 mM iodoacetamide for non-reduced CE or 5% (v/v) beta-mercaptoethanol for reduced in CE-SDS sample buffer (0.5% to 1% SDS, 50–90 mM tris-HCl, pH 9).

## Results and discussion

### Interaction between individual antibodies in the mixture

**Fig 1:**
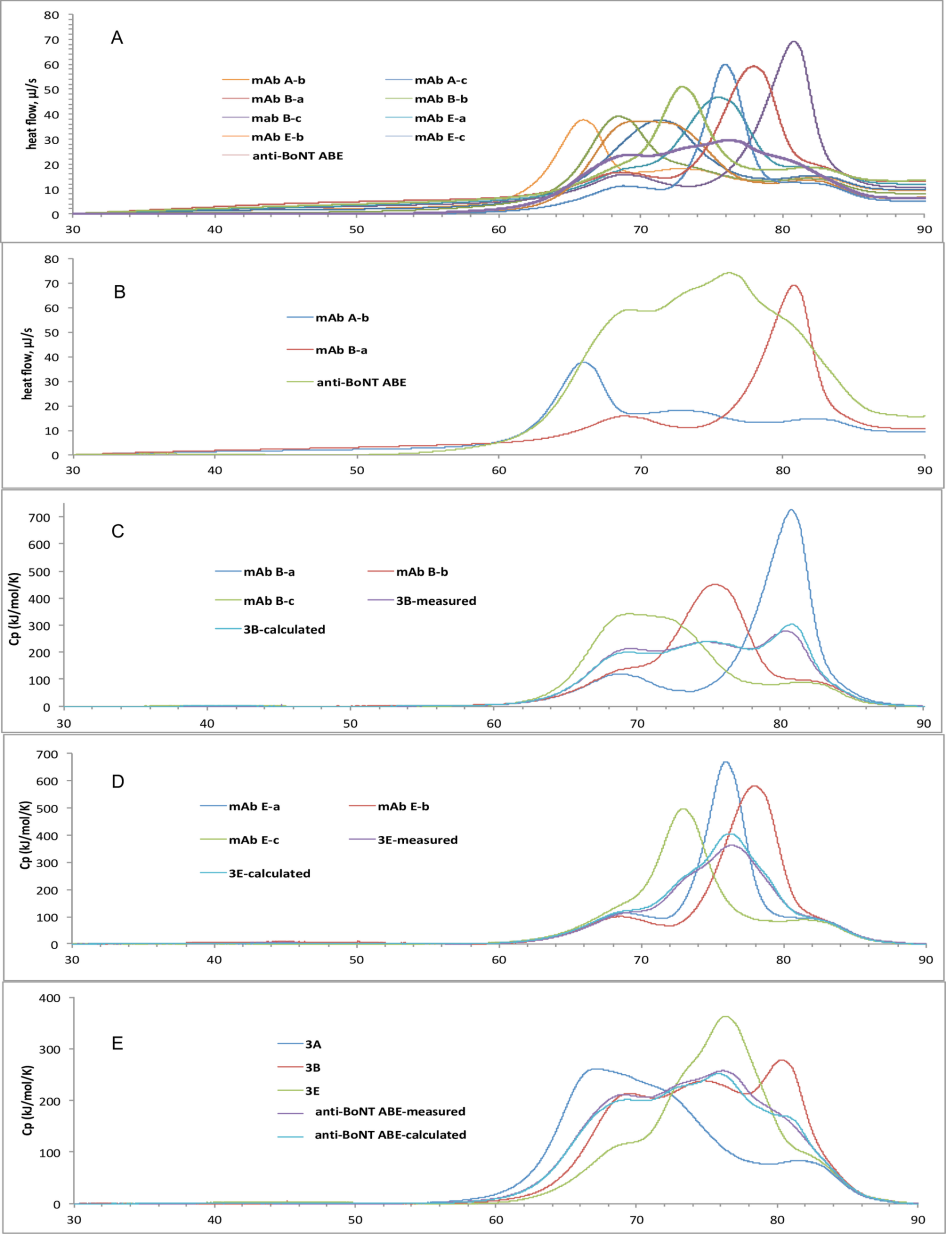
DSC thermograms of anti-BoNT 9ABE and mAbs comprising anti-BoNT 9ABE in the succinate, arginine, Polysorbate formulation, ramp rate 1°C/minute. Results are from single samples. **A.** Thermograms of individual mAbs compared to anti-BoNT 9ABE. Individual mAbs are: mAb A-a, -b, -c, which bind BoNT/A; mAb B-a, -b, -c, which bind BoNT/B; and mAb E-a, E-b, E-c, which bind BoNT/E. **B**. the amplified anti-BoNT 9ABE thermogram overlaid with those of mAbs with the lowest and highest Tm’s–mAb A-b and mAb B-a respectively. Panels C through E compare measured and calculated DSC thermograms normalized for concentration. **C.** Three mAbs that bind to BoNT/B; **D.** Three mAbs that bind BoNT/E. **E.** DSC thermograms of nine-antibody mixture (anti-BoNT 9ABE measured) compared to the sum calculated from the measured thermograms of the nine individual antibodies (anti-BoNT 9ABE calculated) and normalized for concentration. Also shown are thermograms for three mAbs binding BoNT/A (3A), BoNT/B (3B) and BoNT/E (3E).

A challenge for multi-protein formulations is the potential for interaction of component proteins that accelerate aggregation. DSC was used to characterize the unfolding for all nine individual antibodies as well as two-, three- and nine-antibody mixtures in the same formulation under identical conditions. We then compared the measured DSC thermogram of a sample mixture with the thermogram calculated from summation of the individual thermograms of the components, normalized for concentration. DSC thermograms indicated that each antibody in anti-BoNT 9ABE unfolded independently, suggesting that the nine mAbs did not significantly interact. anti-BoNT 9ABE melted over a range encompassing the Tm with the lowest (mAb A-b) and highest (mAb B-a) unfolding temperatures and the melting curves of the mixture closely resemble the sum of the melting of the nine individual mAbs (Fig 1A and 1B). The DSC thermogram for the three-mAb mixtures anti-BoNT 3B (comprising mAb B-a, B-b and B-c) and anti-BoNT 3E (comprising mAb E-a, E-b, E-c) matches closely with the calculated thermograms from the three individual mAbs, as shown in Fig 1C and 1D. The anti-BoNT 9ABE has the same DSC property with respect to the three-mAb mixtures, as shown in Fig 1E. The DSC data indicated that the unfolding of mAbs with lower Tm values did not alter the Tm of other antibodies in the mixture, consistent with minimal interaction among the nine different antibodies in solution.

### Stability after one year of storage at 50°C

**Fig 2:**
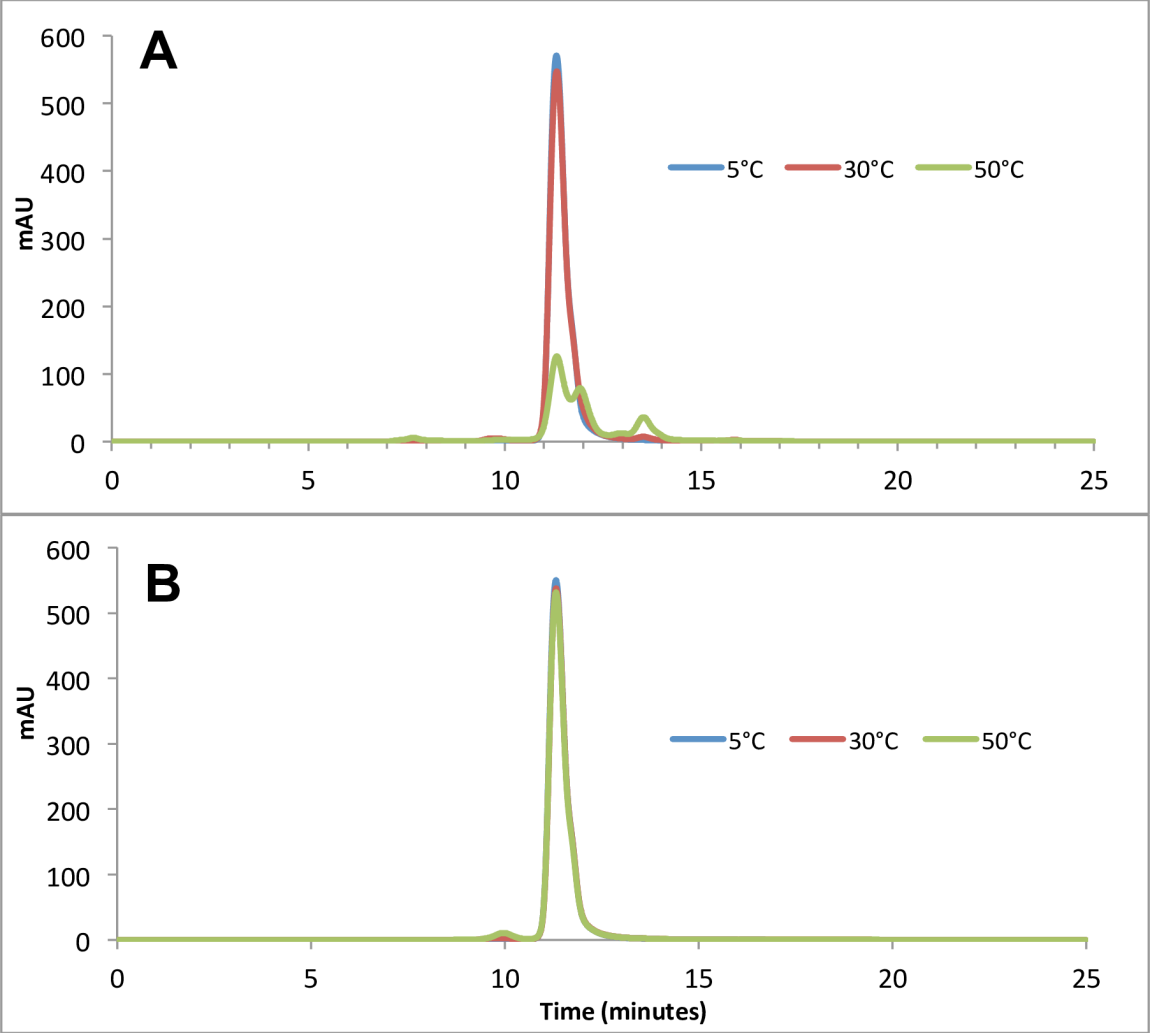
SE-HPLC results of anti-BoNT 9ABE samples stored at 5°C (blue), 30°C (red) and 50°C (green) for six months. **A.** Stored as liquid. **B**. Stored lyophilized.

**Fig 3:**
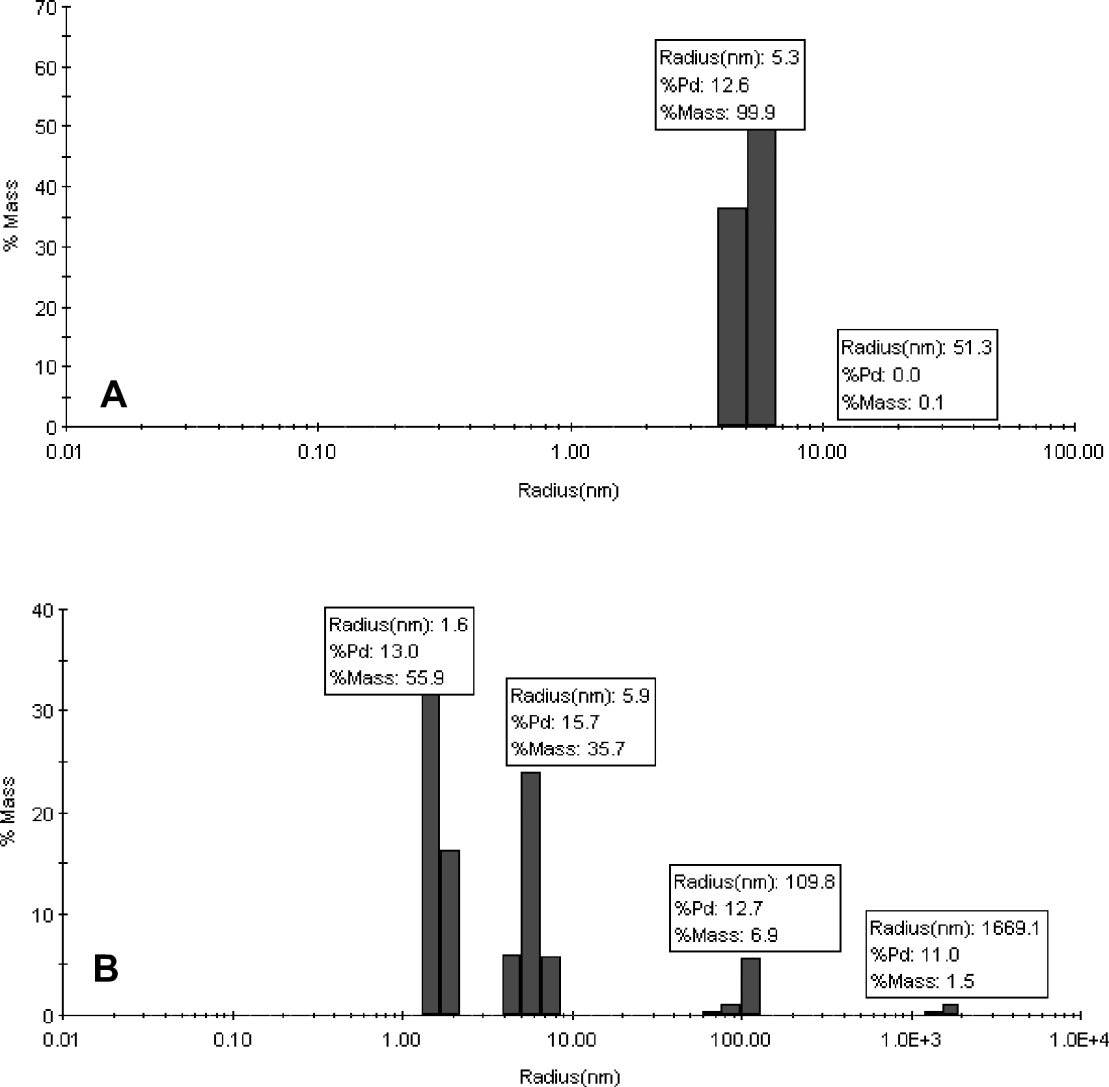
DLS analysis (hydrodynamic radius in nm, % polydispersity, and % mass) of anti-BoNT 9ABE samples stored at 50°C for six months. **A**. Stored lyophilized, **B.** Stored as liquid.

Anti-BoNT 9ABE, was lyophilized at 30 mg/mL in 10 mM sodium succinate, 141 mM L-arginine, 0.01% PS20, pH 6.0. The lyophilized drug product had an acceptable cake structure that was readily reconstituted with water. Lyophilized anti-BoNT 9ABE was analyzed using SE-HPLC and DLS for the presence and quantity of aggregated protein and cleavage. The SE-HPLC elution buffer was chosen to minimize the difference in elution time among antibody monomers so that aggregates or fragments can be quantified without significant overlaps with the monomer peaks. No higher order soluble aggregates or fragments were detected in the lyophilized anti-BoNT 9ABE sample stored at 50°C for up to one year. Chromatograms obtained after 6 months of storage are shown in Fig 2. The soluble portion for the liquid anti-BoNT 9ABE exhibited significant increase in fragments as well as higher order aggregates. The DLS analysis results shown in Fig 3 were consistent with the SEC data. Very few high molecular weight species were detected by DLS in the lyophilized anti-BoNT 9ABE samples stored at 50°C (approximately 0.1% at 6-month and 0.3% at 12 months). Both SEC and DLS indicated that the liquid anti-BoNT 9ABE samples stored at 50°C showed considerable amount of large aggregate species as well as fragments.

**Fig 4:**
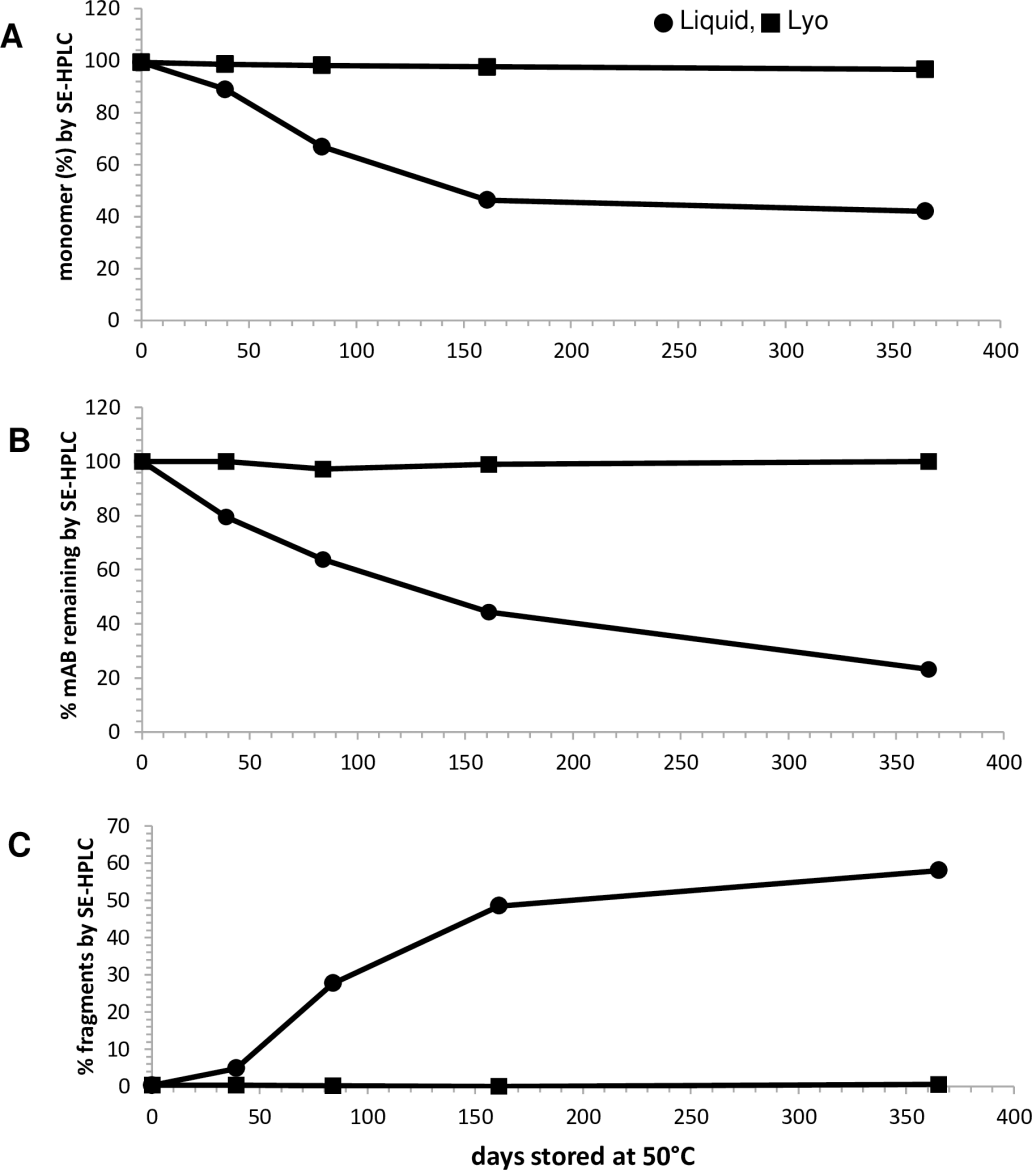
SE-HPLC results for anti-BoNT 9ABE stored at 50°C as lyophilized formulation compared with the liquid formulation for one year. Liquid formulation is shown in circles, lyophilized in squares. **A.** Percent monomer. **B**. Percentage total mAb remaining by peak area relative to initial amount at t = 0. **C.** Percent fragments.

Lyophilized drug product stored at 50°C maintained high percentage of monomer based on SE-HPLC as shown in Fig 4A. The purity for the lyophilized anti-BoNT 9ABE at 50°C decreased only slightly from 99.3 to 96.6% (±0.1%, n = 2) over 12 months due to the increase in dimer species. In contrast, liquid anti-BoNT 9ABE samples contained significant amount of insoluble aggregates at 50°C that had been removed by centrifugation as precipitates, resulting in loss of soluble mAb protein approaching 80% after 12 months (Fig 4B). Liquid anti-BoNT 9ABE samples showed rapid decrease in purity at 50°C due to the formation of fragments (Fig 4C) as well as high molecular weight species.

**Fig 5:**
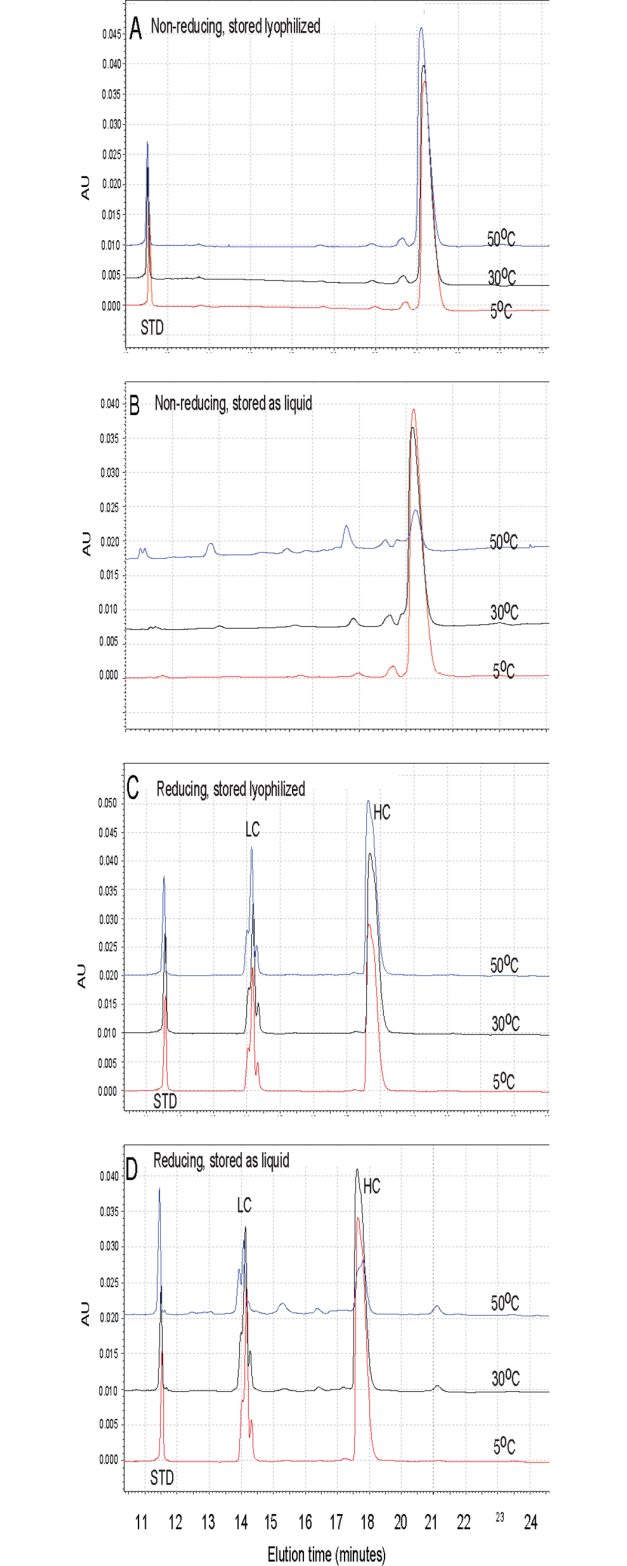
CE-SDS electropherograms of anti-BoNT 9ABE under non-reducing and reducing conditions after storage at 5°C (red), 30°C (black) and 50°C (blue) for six months. **A.** Non-reducing, stored lyophilized. **B.** Non-reducing, stored as liquid. **C.** Reducing, stored lyophilized. **D.** Reducing, stored as liquid. The peak at approximately 11.5 minutes is an internal standard (STD) of 10 kDa in panels **A, C, D**.

Lyophilized anti-BoNT 9ABE shows greater stability than liquid formulation based on CE-SDS, RP-HPLC and WCX-HPLC. Fig 5 shows the non-reduced (Figs 5A and 5B) and reduced (Fig 5C and 5D) CE-SDS electropherograms of the lyophilized and liquid anti-BoNT 9ABE samples stored at three different temperatures (5°C, 30°C and 50°C) for six months. The liquid sample showed rapid degradation as the storage temperature increased, indicated by the appearance of precipitates, non-reducible covalent species, and additional fragment peaks and loss of main peak area, while the lyophilized sample remained relatively un-changed with the storage temperature. The CE-SDS results were consistent with those of SE-HPLC and DLS. The major degradation species in the liquid samples were mostly due to the formation of fragments [32]. Furthermore, significant amount of non-reducible species (peak at approximately 21 minutes) were present in the liquid anti-BoNT 9ABE samples stored at 30°C and 50°C. These non-reducible species were not detected in any of the lyophilized samples, demonstrating the superior stability of the lyophilized anti-BoNT 9ABE product. Minimal high molecular weight species were detected in the non-reduced samples, suggesting that the soluble high molecular weight species detected by the SEC-HPLC and DLS for the 50°C liquid sample were non-covalently associated.

**Fig 6:**
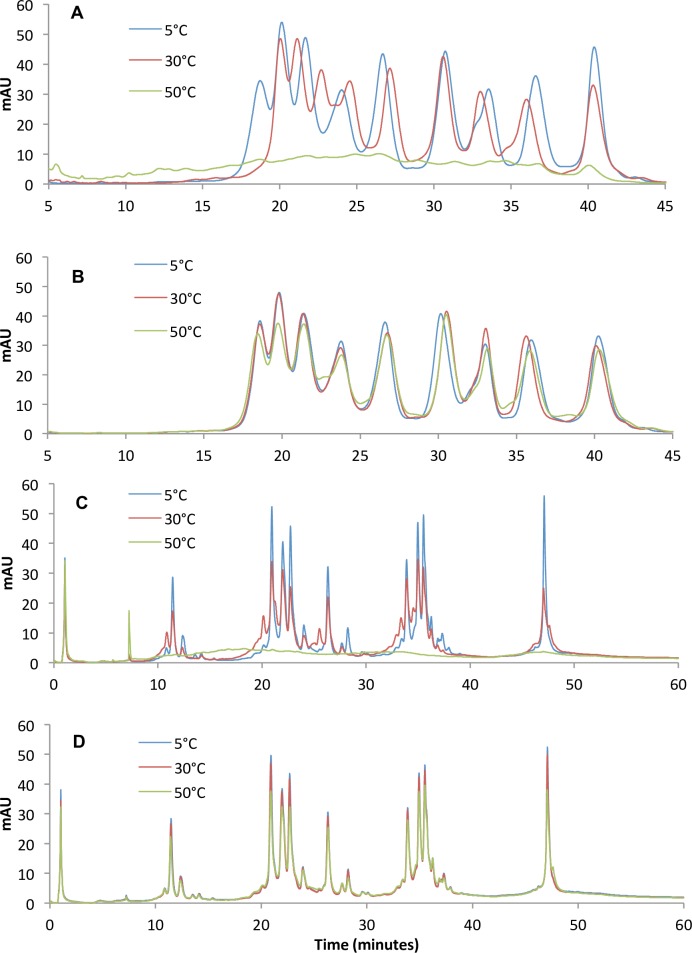
Chromatographic analysis of stored samples. **A and B.** RP chromatograms of anti-BoNT 9ABE stored at 5°C (blue), 30°C (red) and 50°C (green) for six months. **A**. stored as liquid, **B**. stored as lyophilized. Elution order of the peaks was mAb A-c, B-c, E-c, A-b, E-b, A-a, B-a, E-a, and B-b as determined by comparison with retention time of individual mAbs. **C** and **D**. WCX chromatograms of anti-BoNT 9ABE samples stored at 5°C (blue), 30°C (red) and 50°C (green) for six months. The main peaks (>20 mAU) are A-b, E-a, A-a, B-c, B-a, A-c, E-b, B-b, and E-c respectively in elution order. **C.** Stored as liquid. **D.** Stored lyophilized.

Results from RP-HPLC and WCX-HPLC were consistent with results of SE-HPLC, DSC and DLS. As determined by RP-HPLC and WCX-HPLC, anti-BoNT 9ABE stored as liquid exhibited rapid degradation as the storage temperature increased, while the lyophilized counterparts showed relatively unchanged with the temperature, even at 50°C. RP-HPLC chromatograms of anti-BoNT 9ABE stored as liquid (Fig 6A) and lyophilized (Fig 6B) at 5°C, 30°C and 50°C for six months demonstrate the stability of the lyophilized preparations as indicated by loss of peak area as a function increasing temperature. WCX chromatograms of the anti-BoNT 9ABE stored as liquid (Fig 6C) and lyophilized (Fig 6D) at 5°C, 30°C and 50°C for six months also demonstrate stability of the lyophilized samples.

### Antigen binding

**Fig 7:**
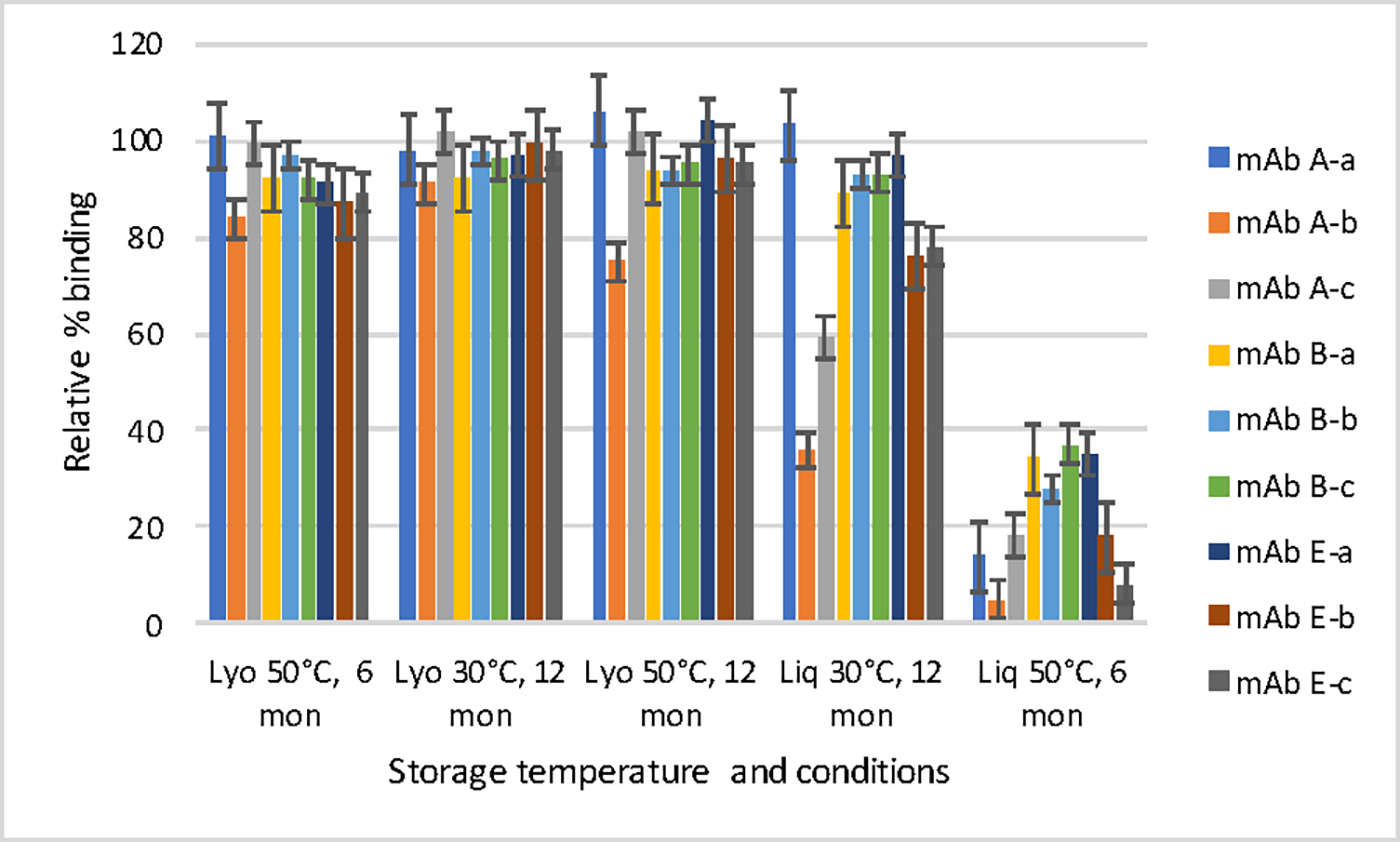
Relative binding of individual antibodies stored at different temperatures and conditions by specific ELISA as a percentage of the value of the individual antibody in anti-BoNT 9ABE stored at 2–8°C in lyophilized state. Error bars indicate standard deviation based on n = 4.

**Fig 8:**
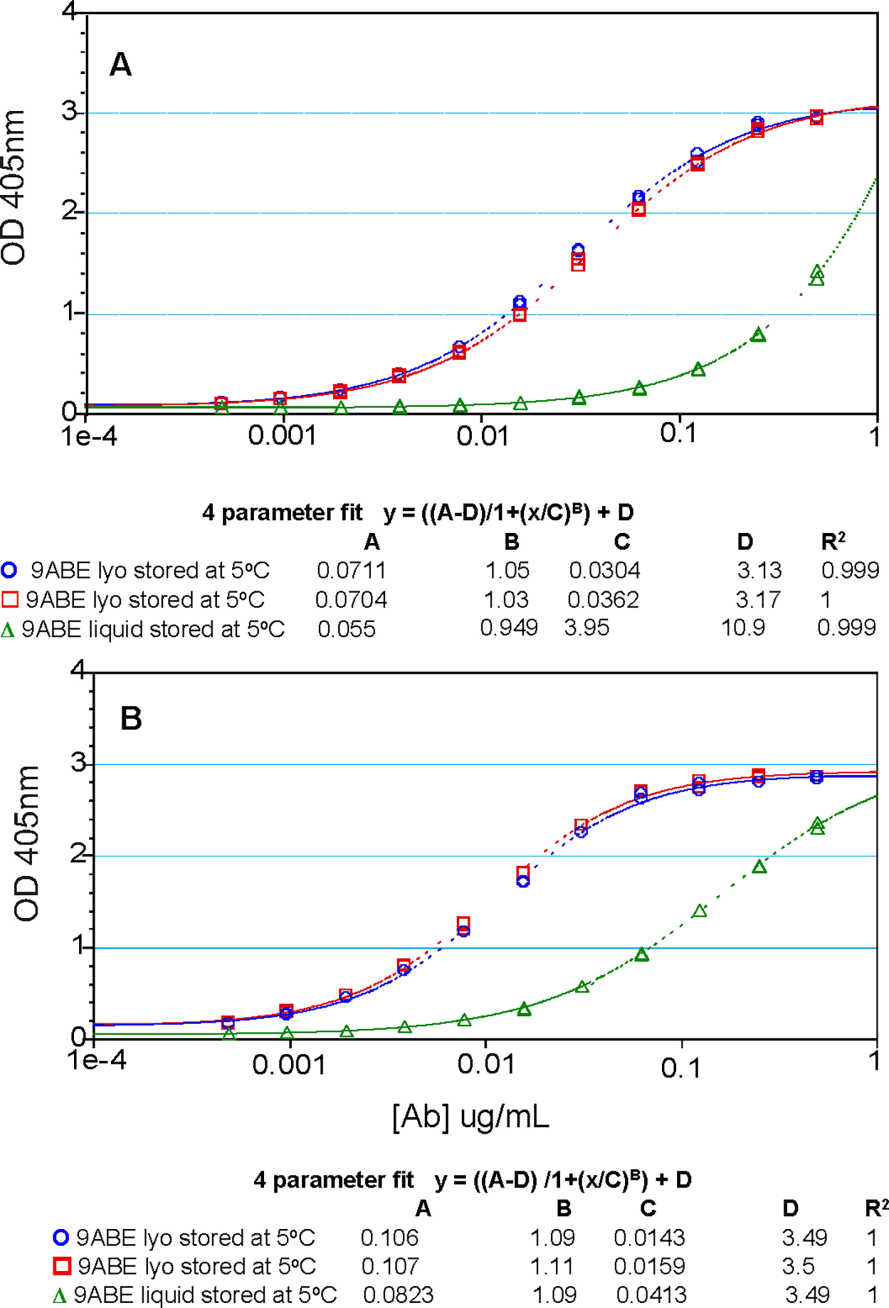
Toxin domain-binding ELISA dose response curves in anti-BoNT 9ABE samples stored under different conditions. Lyophilized anti-BoNT 9ABE stored at 5°C for six months (blue). Lyophilized anti-BoNT 9ABE stored at 50°C for six months (red). Liquid anti-BoNT 9ABE stored at 50°C for six months (green). **A.** domain engineered to bind A-b **B**. domain engineered to bind E-c.

The antigen binding function of each antibody in anti-BoNT 9ABE samples was assessed by ELISA using engineered BoNT domains as previously described [30,31]. The lyophilized anti-BoNT 9ABE stored at 50°C showed almost no loss of antigen binding to respective BoNT domains after six or 12 months (Fig 7). In contrast, storage as liquid resulted in significantly diminished antigen binding by six months. MAbs A-b and E-c showed the greatest reduction in binding ability for the liquid sample as measured by the lower melting temperature by DSC (the dose response curves are shown in Fig 8). ELISA results were consistent with the stability data for individual mAbs. Even after storage for 12 months at 50°C, the individual antibodies in the lyophilized anti-BoNT 9ABE maintained nearly full binding capacity to their respective antigen domains. MAb A-b was found to be one of the least soluble mAbs of the nine and mAb E-c was most prone to cleavage (unpublished data). Since the vast majority of mAbs had precipitated for the liquid sample stored at 50°C for 12 months, no binding ELISA was performed for that sample. All nine of the individual mAbs showed significant decrease (63–95%) in binding capacity following storage at 50°C as a liquid for six months (Fig 7).

## Conclusions

Multiple physicochemical characterization techniques demonstrated that lyophilized anti-BoNT 9ABE has significant stability at room temperature. DSC analysis indicated that the nine individual mAbs in the drug product solution unfolded independently of one another in the study formulation, suggesting that the antibodies did not significantly interact in solution. SE-HPLC analysis demonstrated that the lyophilized drug product showed no detectable soluble higher order aggregates or fragments after storage for 12 months at 50°C. Liquid co-formulation was shown to be impractical for long-term ambient temperature storage because the liquid drug product lost about 80% content as insoluble aggregates and the soluble portion contained mostly fragments after 12 months at 50°C. DLS results were consistent with the SEC results. The superior stability of the lyophilized drug product relative to liquid was confirmed by RP-HPLC and WCX-HPLC, as determined by the least stable antibody in the mixture. The domain-binding ELISA indicated that the nine individual mAbs in the lyophilized anti-BoNT 9ABE drug product showed almost no loss of binding to their respective antigen domains after storage for six months at 50°C, while those stored as liquid showed approximately 80% loss of binding on average, consistent with results of SE-HPLC.

The results presented here demonstrate the feasibility of a stable co-formulation of a large number of antibodies. This is significant for two reasons. Firstly, the stability properties of an ambient-temperature stable biologic drug product makes it ideal for situations where stockpiling and rapid field distribution without need for a cold chain, such as for biodefense or infectious disease outbreak. Secondly, the expected safety properties of fully human oligoclonal antibodies targeting toxins or pathogens makes them ideal in the context of biodefense situations or infectious disease outbreaks where there were multiple confirmed and suspected cases and rapid treatment in the absence of the ability to rapidly confirm diagnosis is indicated.
